# Preeclampsia Risk in Obese Pregnant Women without Diabetes Mellitus: a Meta-Analysis Comparing Metformin versus Control Group

**DOI:** 10.61622/rbgo/2026rbgo46

**Published:** 2026-08-20

**Authors:** Bianca Cardoso Lopes, Nathália Maria Ladeira de Oliveira, Vinícius Diniz Cedro Araújo, Fabyola Jorge Cruz

**Affiliations:** 1 Universidade Federal de Ouro Preto Medical School Minas Gerais Brasil Medical School, Universidade Federal de Ouro Preto, Ouro Preto - Minas Gerais. Brasil

**Keywords:** Preeclampsia, Risk, Obese, Diabetes Mellitus, Pregnancy

## Abstract

**Objective:**

To evaluate the impact of metformin on reducing the risk of pre-eclampsia in obese pregnant women without diabetes.

**Sources of Data:**

Following PRISMA guidelines, Scopus, Embase, PubMed (MEDLINE), Cochrane Database of Systematic Reviews, LILACS, SciElo, and Web of Science were searched for articles published between 2014 and April 2024. Search terms: ("Metformin" OR "Biguanide" OR "Glucophage" OR "dimethylbiguanide") AND ("pre-eclampsia" OR "preeclampsia") AND ("obese pregnant women" OR "obesity in pregnancy" OR "maternal obesity" OR "pregnancy" OR "obesity").

**Study Selection:**

From 2022 studies identified, three randomized controlled trials comparing metformin with placebo or standard care in obese pregnant women without diabetes were included. Primary outcomes were pre-eclampsia incidence; secondary outcomes included gestational hypertension, gestational diabetes, preterm birth, cesarean delivery, postpartum hemorrhage, and NICU admission.

**Data collection:**

Data were extracted, and odds ratios (OR) with 95% confidence intervals (CI) were calculated. Heterogeneity was assessed using I^2^ statistics.

**Data Synthesis:**

Metformin did not significantly reduce PE risk (OR: 0.42; 95% CI: 0.10–1.68; p=0.22; I^2^=81%). Secondary outcomes showed no significant differences. Subgroup analysis excluding a methodological outlier suggested potential benefit (OR: 0.20; 95% CI: 0.10–0.38; p<0.00001; I^2^=0%). Metformin alone may not effectively prevent PE in these women, but efficacy could improve with lifestyle modifications. Study heterogeneity highlights the need for larger, rigorous trials.

**Conclusions:**

Metformin should not be routinely recommended for pre-eclampsia prevention in obese pregnant women without diabetes until robust evidence from further research supports its efficacy.

**Prospero registry:** CRD42024611538.

## Introduction

Hypertensive disorders of pregnancy (HDP) encompass a complex spectrum of conditions, with preeclampsia (PE) and eclampsia representing the most severe manifestations. These disorders significantly contribute to maternal and neonatal morbidity and mortality worldwide, particularly in low- and middle-income countries.^(1)^ PE, characterized by the development of hypertension and target organ damage after 20 weeks of gestation, is associated with severe adverse outcomes, including placental insufficiency, intrauterine growth restriction (IUGR), and prematurity.^(2)^ Hence, it is crucial to study ways to reduce the risk of PE. In this context, metformin is a drug of interest. Although metformin is extensively studied in the context of gestational diabetes, its direct impact on the prevention of PE in non-diabetic women remains controversial. Previous studies on the relationship between metformin and PE have reported conflicting results, primarily limited by small sample sizes and methodological heterogeneity.^(1,3)^ An updated systematic review and meta-analysis, utilizing data from randomized clinical trials and focusing on obese women without type 2 Diabetes Mellitus (DM2), is essential to clarify this issue and potentially offer a new preventive approach for PE.

Therefore, this study aims to fill a gap in the literature based on the following PICO question: among obese pregnant women without Diabetes Mellitus (P), does metformin administration during pregnancy (I), compared with no metformin use (C), reduce the incidence of preeclampsia (O)? In this way, we aim to provide evidence-based management strategies to improve maternal and neonatal health outcomes.

## Methods

This systematic review with meta-analysis was registered in the International Prospective Register of Systematic Reviews (PROSPERO) with the protocol number CRD42024611538. The Cochrane Collaboration recommendations for systematic reviews and meta-analyses were used, and the reporting of results followed the PRISMA (Preferred Reporting Items for Systematic Reviews and Meta-Analyses) guidelines.

### Eligibility Criteria

The eligibility criteria were: (1) randomized clinical trials; (2) studies comparing metformin with a control group; (3) pregnant patients with obesity and without DM; (4) studies reporting at least one of the clinical outcomes of interest. The exclusion criteria were: (1) preclinical studies; (2) studies without a control group; or (3) crossover study designs.

### Search Methods

The systematic review and meta-analysis were conducted according to the recommendations of the Cochrane Collaboration and the Preferred Reporting Items for Systematic Reviews and Meta-Analysis (PRISMA) guidelines.^(4)^ A systematic search was performed in the databases Scopus, Embase, PubMed (MEDLINE), Cochrane Database of Systematic Reviews, LILACS (Latin American and Caribbean Health Sciences Literature), SciElo, and Web of Science, using the following descriptors: ("Metformin" OR "Biguanide" OR "Glucophage" OR "dimethylbiguanide") AND ("pre-eclampsia" OR "preeclampsia") AND ("obese pregnant women" OR "obesity in pregnancy" OR "maternal obesity" OR "pregnancy" OR "obesity"). For the construction of these search descriptors, the Medical Subject Headings (MeSH) and Emtree databases were consulted. Additionally, the following Portuguese descriptors were used in LILACS and Scielo: ("Metformina" OR "Biguanida") AND (Pré-eclâmpsia) AND ("Obesidade materna"). The complete search strings for all databases can be found in the Supplementary Material (Table S1). For the descriptors construction, the Health Sciences Descriptors (DECS) were used. No date or language restrictions were applied and the last search was performed in April 2024. Moreover, the references of included studies and systematic reviews were evaluated for additional studies.

### Outcomes

The primary outcome of interest in this meta-analysis was preeclampsia. Other outcomes included postpartum hemorrhage (blood loss greater than 1000 mL), development of gestational hypertension, development of gestational diabetes mellitus, prematurity, the need for neonatal intensive care unit (NICU) admission, and cesarean delivery.

### Quality Assessment

The Cochrane Collaboration tool^(5)^ was used to assess the risk of bias in randomized trials and to evaluate the quality of individual randomized studies. The risk of bias assessment was independently conducted by three authors (B.L., N.O., and F.C). Publication bias was assessed with a funnel plot analysis for the primary outcome to evaluate the symmetrical distribution of trials with similar weights.

### Data Analysis

All identified articles were systematically evaluated using the inclusion and exclusion criteria. Article screening was conducted blindly by two reviewers using the RAYYAN tool. Full-text article selection and data extraction were independently performed by two reviewers, and discrepancies were reviewed by a third reviewer. The treatment effects for binary outcomes were compared using pooled odds ratios (OR) with 95% confidence intervals (CI95%). Heterogeneity was assessed with Cochran's Q test and I^2^ statistics; p-values < 0.10 and I^2^ > 25% were considered significant for heterogeneity. A fixed-effect model was used for outcomes with p > 0.10 and I^2^ < 25% (low heterogeneity). For outcomes with high heterogeneity, the DerSimonian and Laird random-effects model was used. Statistical analysis was conducted using Review Manager (Nordic Cochrane Centre, The Cochrane Collaboration, Copenhagen, Denmark), and RStudio version 4.4.2 software was used to create L’Abbé plots and "Leave-one-out" plots if heterogeneous results were identified.

## Results

### Study Selection and Baseline Characteristics

A total of 2022 studies were found in the bibliographic search (Figure 1). After removing duplicate records and studies based on the title/abstract review, 20 articles were obtained. These were meticulously reviewed according to the inclusion and exclusion criteria, resulting in the inclusion of 4 studies. However, the article by Balani et al. (2017)^(6)^ is a secondary analysis of the "MOP trial" previously described by Syngelaki et al. (2016). To avoid sample overlap, data from 3 studies^(7,8,10)^ were used, including 1206 non-overlapping patients, of whom 599 (49.7%) used metformin. The characteristics of the included articles are available in Table 1. The doses of metformin used in each study are available in Table 2.

**Figure 1:**
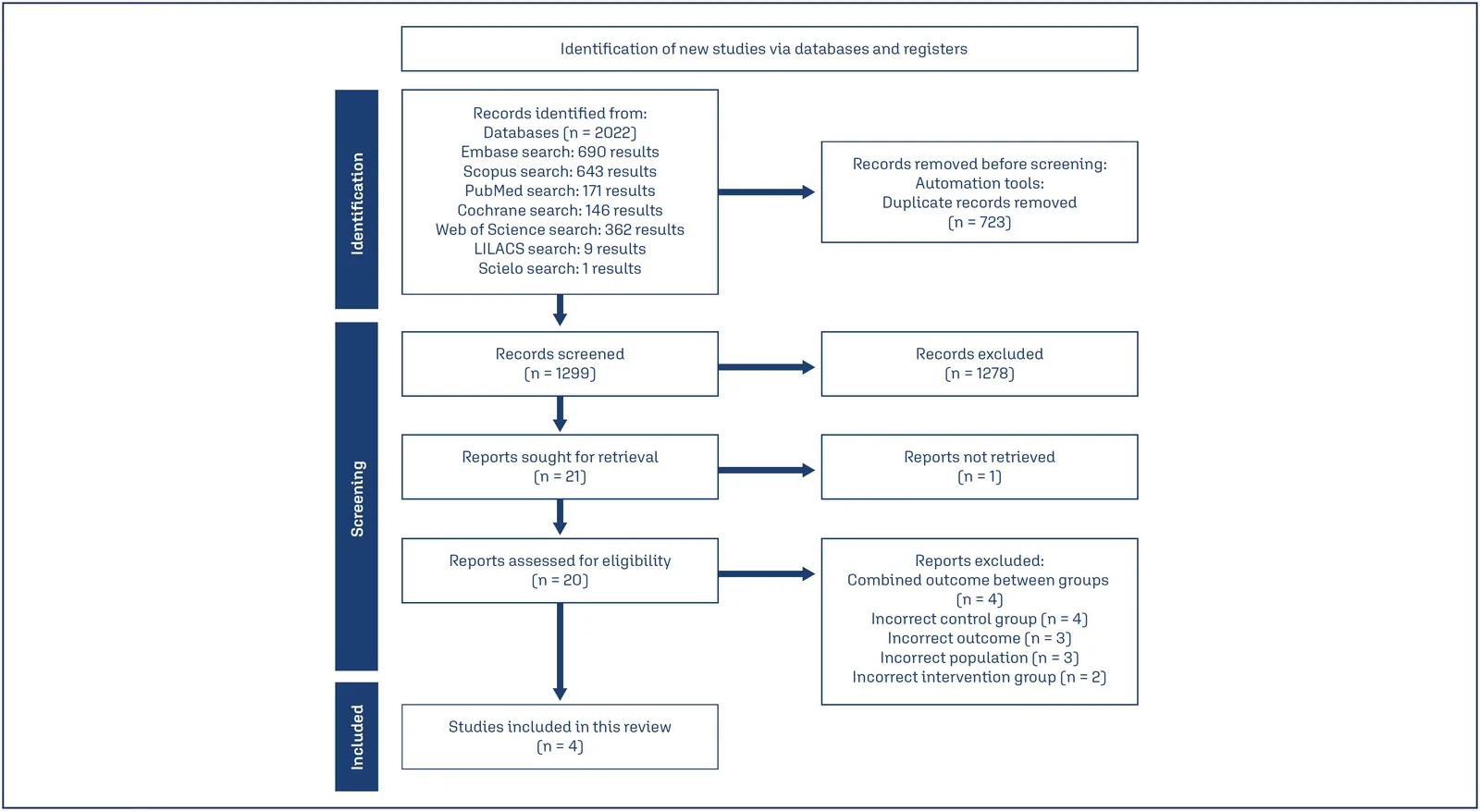
PRISMA Flow Diagram of Study Screening and Selection

Source: the authors

**Table 1:** Baseline characteristics of included studies

	Chiswick 2015		Syngelaki 2016		Nascimento 2020	
	Control Group	Intervention Group	Control Group	Intervention Group	Control Group	Intervention Group
Number of Participants	223	226	198	202	186	171
Average Maternal Age (in years)	28.9	28.7	30.8**	32.9**	29.6*	28,6
Body Mass Index at Study Entry	37.7	37.8	38.4	38.6	37.4	37.2
Average Gestational Age at Study Entry (in weeks)	14.1	14.1	14.9**	15.1**	11.3	11.5
Previous Hypertensive Patients	7	10	17	13	NI	NI
Patients with Polycystic Ovary Syndrome	21	28	18	26	NI	NI
Smokers	31	40	21	15	NI	NI

* Mean

** Median

**Table 2:** Dose of metformin used by the intervention group in each study

Study	Dose of Metformin Used by the Intervention Group
Chiswick 2015	Metformin was initiated at a daily dose of 500 milligrams (mg) at the first week and escalated by one tablet a day each week over 5 weeks, to reach either the maximum tolerable dose or the maximum dose of 2500 mg.
Syngelaki 2016	Metformin was initiated at a daily dose of 1000 mg in the first week, and the dose was increased by 500 mg per week to reach the maximum dose of 3000 mg (week 5). Participants with serious side effects were asked to continue taking the maximum tolerated dose.
Nascimento 2020	500 milligrams breakfast and at dinner (1000 milligrams daily).

### Outcome Analysis

Regarding the primary outcome, the data suggest that there is no significant correlation between the use of metformin and the reduction in the likelihood of developing preeclampsia in obese women without diabetes. However, there appears to be severe heterogeneity among the studies included in the meta-analysis [OR = 0.42; 95% CI: 0.10-1.68; p = 0.22; I^2^ = 81%] (Figure 2). Similarly, there was no significant difference between the groups for the outcome of gestational diabetes development [OR = 0.85; 95% CI: 0.58-1.22; p = 0.37; I^2^ = 0%] (Figure 2) and cesarean deliveries [OR = 0.66; 95% CI: 0.40-1.10; p = 0.11; I^2^ = 78%] (Figure 2).

**Figure 2:**
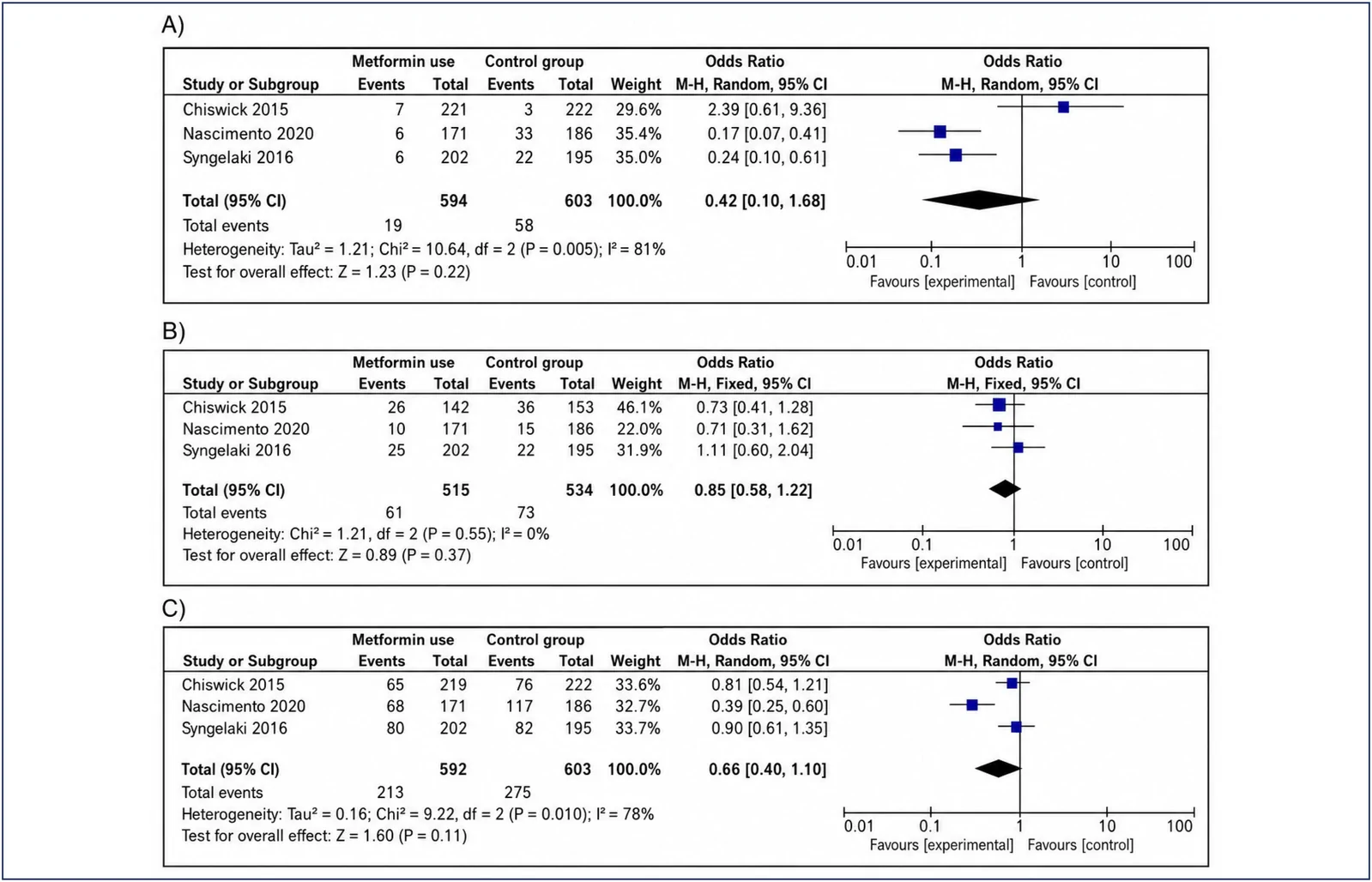
A) There is no significant difference in the occurrence of preeclampsia between the groups using metformin and the placebo group (p=0.22). There is substantial heterogeneity among the included studies (I^2^ = 81%). B) There is no significant difference in the outcome of the development of gestational diabetes between the groups using metformin and the control group (p=0.37). C) There is no significant difference in the incidence of cesarean deliveries between the groups using metformin and the control group (p=0.11).

Only the studies by Chiswick 2015 and Syngelaki 2016^(7,8)^ provide data on postpartum hemorrhage with the use of metformin. Although there is homogeneity in the results regarding this maternal outcome, no significant statistical association was observed between the use of metformin and the studied population for this outcome [OR = 1.05; 95% CI: 0.66-1.69; p = = 0.83; I^2^ = 0%] (Figure 3). These same studies also cite the development of hypertension as a maternal outcome with homogeneous data, showing that there is no significant correlation between the use of metformin and hypertension [OR = 1.26; 95% CI: 0.75-2.14; p-value = 0.38; I^2^ = 0%] (Figure 3).

**Figure 3:**
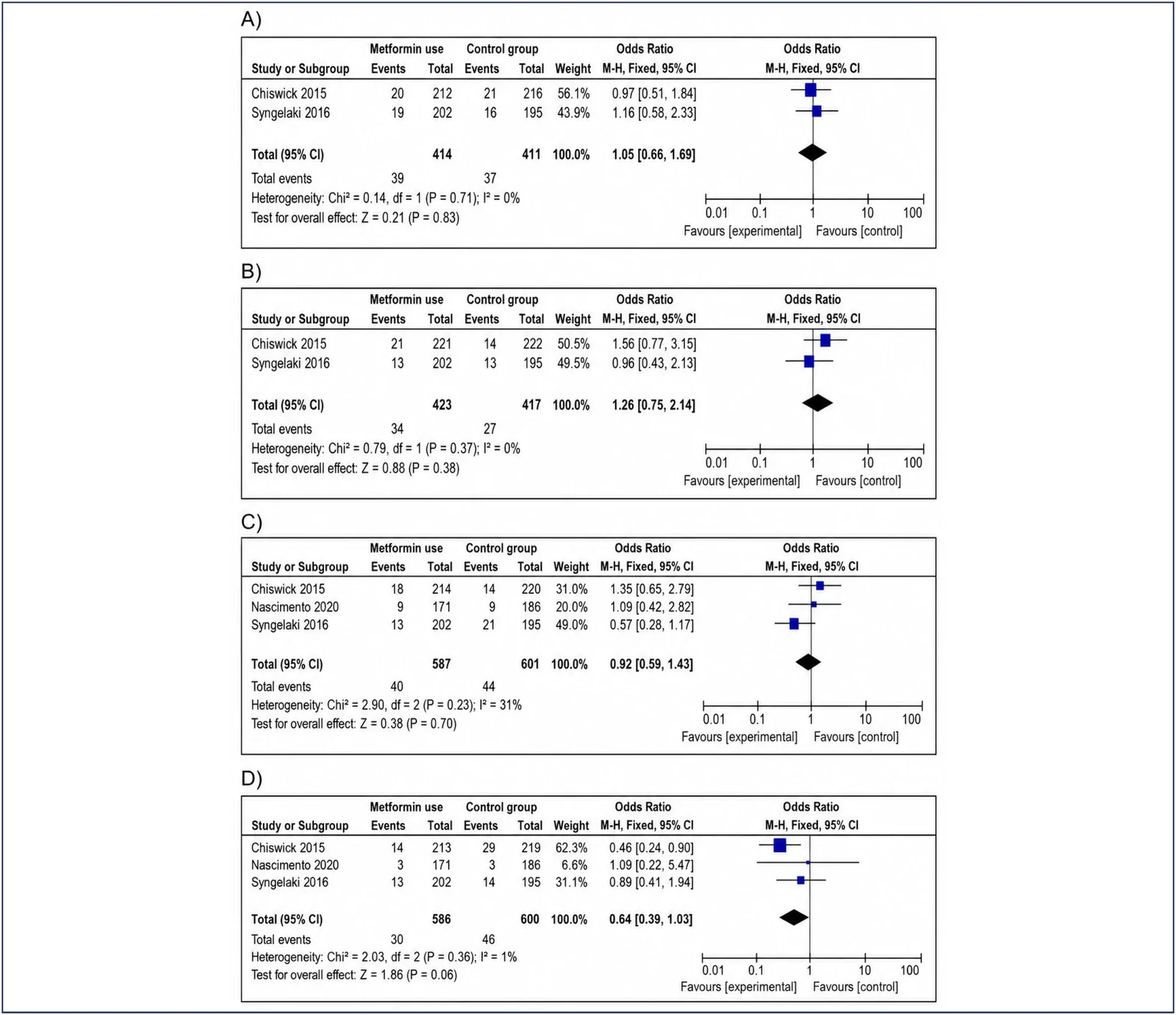
A) There is no significant difference in the incidence of postpartum hemorrhage (considering losses greater than 1000 ml) between the groups using metformin and the control group (p=0.83). B) There is no significant difference in the outcome of the development of gestational hypertension between the groups using metformin and the control group (p=0.38). C) There is no significant difference in the incidence of prematurity between the groups using metformin and the control group (p=0.70). D) There is no significant difference in the neonatal intensive care unit (NICU) admission rate between the groups using metformin and the control group (p=0.06).

Regarding fetal outcomes, data analysis from the three studies suggests that there is no statistically significant difference between the intervention group and placebo concerning prematurity. Even though there is observed heterogeneity among the three studies, it is not significant [OR = 0.92; 95% CI: 0.59-1.43; p = 0.70; I^2^ = 31%] (Figure 3). Regarding the need for neonatal intensive care unit (NICU) admission, although there is consistency among the studies and a reduction in odds, it is not significant [OR = 0.64; 95% CI: 0.39-1.03; p = 0.06; I^2^ = 1%] (Figure 3).

A more detailed analysis shows a severe degree of heterogeneity among the included studies. The Baujat plot (Figure 4) highlights the article by Chiswick (2015)^(7)^ as the main contributor to this heterogeneity, significantly influencing the final result. This outcome can also be observed in the L’Abbé plot (Figure 4), identifying the same article as an "outlier."

**Figure 4:**
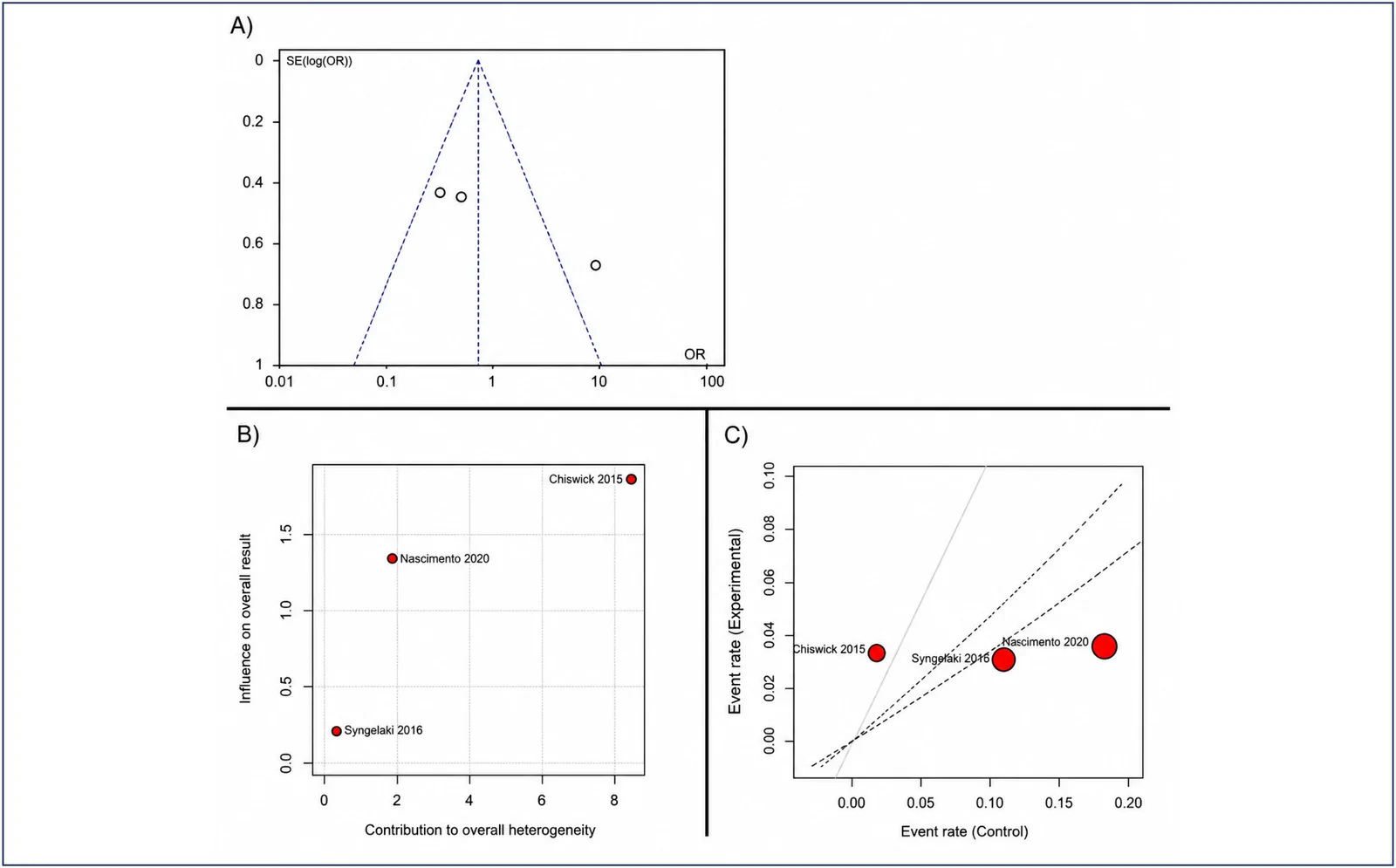
A) Funnel Plot for the Assessment of Study Weights. B) Baujat Plot for the Approach to Heterogeneity and Influence of Studies. C) L’Abbé Plot for the Approach to Heterogeneity and Individual Study Results.

Given this, when excluding Chiswick's study from the analysis, leaving only the studies by Syngelaki (2016) and Nascimento (2020)^(8,10)^, the incidence of preeclampsia becomes statistically significant, favoring the use of metformin [OR = 0.20; 95% CI: 0.10-0.38; p < 0.00001; I^2^ = 0%] (Figure 5), which justifies the observed heterogeneity when the analysis considers all three studies. This underscores the importance of considering methodological and contextual factors that may influence the findings.

**Figure 5:**
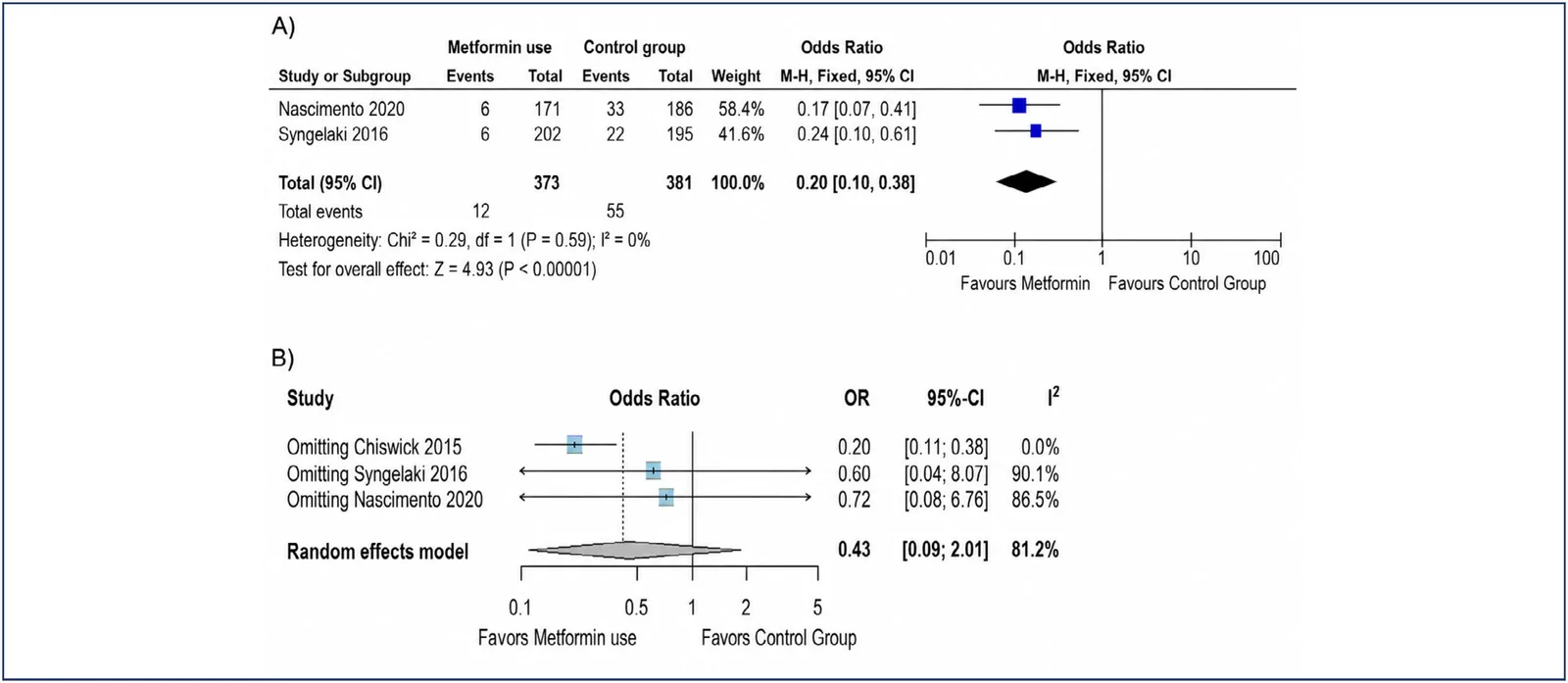
A) Subgroup analysis of articles where participants were instructed on lifestyle changes. A significant reduction in the recurrence of preeclampsia is observed in the group using metformin compared to the control group D(OR = 0.20; p<0.00001). OR = Odds Ratio. B) "Leave one out" showing that excluding Chiswick et al. 2015 yields homogeneous and significant results. OR = Odds Ratio.

### Risk of Bias

The Cochrane Collaboration tool (Risk of Bias 2 - RoB 2) was used to assess the quality of this meta-analysis. No study was considered high risk (Table 3). In the funnel plot analysis (Figure 4), the studies occupied an asymmetric distribution according to weight, but given the small number of studies, it is not possible to decisively infer publication bias.^(9)^

**Table 3:** Cochrane risk of bias assessment results for included studies, showing a low risk of bias in included studies.

Study	Bias from randomization process	Bias due to deviations from intended interventions	Bias due missing outcome data	Bias in measurement of the outcomes	Bias in selection of the reported result	Overall risk of bias
Chiswick 2015	Low	Some concerns	Low	Low	Low	Low
Syngelaki 2016	Low	Low	Low	Low	Low	Low
Nascimento 2020	Low	Low	Low	Low	Low	Low

## Discussion

In this systematic review and meta-analysis, no significant difference was observed between the use of metformin and the control group for the outcome of preeclampsia.

As previously described, the substantial heterogeneity among the studies may be related to Chiswick's methodological and contextual factors. One key factor identified was the lack of dietary and physical activity guidance in Chiswick's study, which significantly impacted the aggregated results. When data from this study were excluded from the analysis, leave-one-out (Figure 5), a significant favorable association between metformin use and the reduced risk of preeclampsia was observed in the remaining studies, especially in contexts where participants received additional lifestyle-related interventions. It is necessary to consider that physical exercise alone may be responsible for the positive outcomes observed.^(11)^ But, this may also suggest that metformin could have a synergistic effect when combined with strategies aimed at improving metabolic and cardiovascular health during pregnancy.

The absence of effect on secondary outcomes, such as the development of gestational hypertension, gestational diabetes, prematurity, and NICU admission, cesarean delivery, and postpartum hemorrhage, raises the hypothesis that the impact of metformin may be limited to specific patient subgroups or depend on additional factors, such as the timing of therapy initiation and adherence to other concomitant interventions. Stratified analysis of these outcomes in different populations is necessary.

Additionally, bias analysis and the small number of included studies represent significant limitations. The asymmetry in the funnel plot, although influenced by the low sample size, may indicate publication bias or variations in study quality. Thus, it is crucial for future clinical trials to be designed with larger sample sizes, standardized protocols, and the inclusion of complementary interventions, such as lifestyle changes.

Although the overall data do not show a significant difference, targeted analysis suggests a potential benefit of metformin in specific contexts, particularly when associated with interventions aimed at optimizing maternal metabolism, especially when analyzing the pathophysiology of PE. This is multifactorial and involves a complex interaction between immunological changes, angiogenic factors, oxidative stress, and endothelial dysfunction.^(12)^

Impaired trophoblastic invasion in early pregnancy results in inadequate remodeling of the spiral arteries, leading to placental ischemia and increased local oxidative stress. This promotes the release of anti-angiogenic factors into the maternal circulation, such as placental growth factor (PlGF) and soluble fms-like tyrosine kinase-1 (sFlt-1), which exacerbate systemic endothelial dysfunction, contributing to hypertension and target organ damage.^(13)^

Metformin, a biguanide widely used in the management of type 2 diabetes mellitus (DM2), exerts its primary effect by reducing hepatic glucose production and improving insulin sensitivity.^(14)^ However, recent evidence suggests that its effects go beyond glycemic metabolism. Metformin has antioxidant and anti-inflammatory properties, capable of modulating endothelial dysfunction, a central feature of PE. Furthermore, the drug can improve mitochondrial function and reduce the production of reactive oxygen species (ROS), processes involved in the pathophysiology of PE.^(15)^ In vitro and in vivo studies indicate that metformin can attenuate the inflammatory response and oxidative stress induced by placental ischemia, suggesting its potential use in the context of PE.^(16)^

Obese women have an elevated risk of developing PE due to the interaction between chronic inflammation, insulin resistance, and changes in metabolic homeostasis. Excess adipose tissue is a source of pro-inflammatory cytokines, such as IL-6 and TNF-α, which aggravate endothelial dysfunction and increase oxidative stress.^(17)^ In this context, metformin emerges as a potentially beneficial intervention, as it can mitigate these pathological pathways by modulating lipid and inflammatory metabolism. However, a previous meta-analysis with fewer included studies (smaller n) described a significant reduction in maternal weight gain in obese pregnant women without DM2 but did not observe the same effect on birth weight or preeclampsia reduction, as seen in the present study.^(18)^

In this sense, although it is not possible to build more robust evidence on the use of metformin in pregnant, obese, and non-diabetic patients in reducing PE through this systematic review and meta-analysis alone, it is understood that more multicenter randomized clinical trials with larger sample sizes and greater scale are fundamental to validate findings and provide clearer guidelines for clinical practice. Thus, the present article, based on the results found, is limited in directing future studies to investigate whether, in fact, the association of metformin with physical activity would have a positive effect.

It is important to consider that low-dose aspirin is currently the standard of care for the prevention of preeclampsia (PE) in high-risk pregnant women. However, only one of the included studies explicitly reported whether participants were also taking aspirin. Only the study by Nascimento addressed this issue. The authors noted that the incidence of large-for-gestational-age (LGA) newborns was higher in the group treated with aspirin compared to the placebo group (40.2% vs. 26.6%, *P* = 0.005).^(10)^ Further studies are needed to systematically evaluate the concomitant use of aspirin in trials assessing the effectiveness of metformin for the prevention of PE, as this variable may significantly influence outcomes and interpretations.

This meta-analysis differs from previous works by presenting a larger sample size of women, adding a significant article to the meta-analysis, and considering that the last work was published seven years ago. Additionally, previous articles compared placebo and metformin, while this work compares placebo associated with lifestyle changes and the use of metformin associated with lifestyle changes.

## Conclusion

The results of this systematic review and meta-analysis suggest that, overall, the use of metformin does not result in a statistically significant reduction in the incidence of preeclampsia in obese women without type 2 Diabetes mellitus. However, a more detailed analysis indicates that heterogeneity among the studies directly influences the findings, highlighting the impact of additional interventions, such as dietary and physical activity guidance, in enhancing the effects of metformin.

Also, in light of current clinical guidelines recommending low-dose aspirin for PE prevention, future research should explore the combined use of metformin and aspirin in high-risk pregnant women. Investigating the potential synergistic or adverse effects of this combination may provide more effective and safer strategies for the prevention of preeclampsia.

Given the limited number of available studies and the presence of significant heterogeneity, this work underscores the need for future randomized clinical trials with greater methodological rigor and robust sample sizes to assess the impact of metformin in different contexts. Until new data are generated, the use of metformin in the preventive management of preeclampsia should be evaluated cautiously and within an integrated approach that includes lifestyle changes. Such findings may provide important insights for developing more effective strategies in maternal and infant health management.

## Data Availability

The research data are described in the article presented.
