# Supplementary material for: Preeclampsia Risk in Obese Pregnant Women without Diabetes Mellitus: a Meta-Analysis Comparing Metformin versus Control Group
Source: Rev Bras Ginecol Obstet. 2026 Aug 20;48:e-rbgo46. doi: 10.61622/rbgo/2026rbgo46 (PMC13690377; doi:10.61622/rbgo/2026rbgo46)
Supplement: Supplementary file 1 [file 1806-9339-rbgo-48-e-rbgo46-Suppl01.pdf]

Supplementary Material

Table S1. Search strategies

Pubmed
("Metformin" OR "Biguanide" OR "Glucophage" OR "dimethylbiguanide") AND ("pre-eclampsia" OR "preeclampsia") AND ("obese pregnant women" OR "obesity in pregnancy" OR "maternal obesity" OR "pregnancy" OR "obesity")

Embase
("Metformin" OR "Biguanide" OR "Glucophage" OR "dimethylbiguanide") AND ("pre-eclampsia" OR "preeclampsia") AND ("obese pregnant women" OR "obesity in pregnancy" OR "maternal obesity" OR "pregnancy" OR "obesity")

Cochrane Database of Systematic Reviews:
("Metformin" OR "Biguanide" OR "Glucophage" OR "dimethylbiguanide") AND ("pre-eclampsia" OR "preeclampsia") AND ("obese pregnant women" OR "obesity in pregnancy" OR "maternal obesity" OR "pregnancy" OR "obesity")

Web of Science:
("Metformin" OR "Biguanide" OR "Glucophage" OR "dimethylbiguanide") AND ("pre-eclampsia" OR "preeclampsia") AND ("obese pregnant women" OR "obesity in pregnancy" OR "maternal obesity" OR "pregnancy" OR "obesity")

Scopus:
("Metformin" OR "Biguanide" OR "Glucophage" OR "dimethylbiguanide") AND ("pre-eclampsia" OR "preeclampsia") AND ("obese pregnant women" OR "obesity in pregnancy" OR "maternal obesity" OR "pregnancy" OR "obesity")

LILACS:
First screening: ("Metformin" OR "Biguanide" OR "Glucophage" OR "dimethylbiguanide") AND ("pre-eclampsia" OR "preeclampsia") AND ("obese pregnant women" OR "obesity in pregnancy" OR "maternal obesity" OR "pregnancy" OR "obesity")
Second screening: ("Metformina" OR "Biguanida") AND (Pré-eclâmpsia) AND ("Obesidade materna")

SciElo:
First screening: ("Metformin" OR "Biguanide" OR "Glucophage" OR "dimethylbiguanide") AND ("pre-eclampsia" OR "preeclampsia") AND ("obese pregnant women" OR "obesity in pregnancy" OR "maternal obesity" OR "pregnancy" OR "obesity")
Second screening: ("Metformina" OR "Biguanida") AND (Pré-eclâmpsia) AND ("Obesidade materna")
